# Avoidable costs of stenting for aortic coarctation in the United Kingdom: an economic model

**DOI:** 10.1186/s12913-017-2215-2

**Published:** 2017-04-10

**Authors:** Maximilian Salcher, Alistair Mcguire, Vivek Muthurangu, Marcus Kelm, Titus Kuehne, Huseyin Naci, CARDIOPROOF, Edwin Morley-Fletcher, Titus Kuehne, Anja Hennemuth, David Manset, Alistair Mcguire, Gernot Plank, Olivier Ecabert, Giacomo Pongiglione, Vivek Muthurangu

**Affiliations:** 1 LSE Health and Social Care, Cowdray House; London School of Economics and Political Science, Houghton Street, London, WC2A 2AE UK; 2 UCL Institute of Cardiovascular Science & Great Ormond Street Hospital for Children, Great Ormond Street Hospital, London, UK; 3 Department of Paediatric Cardiology and Congenital Heart Diseases, Deutsches Herzzentrum Berlin, Berlin, Germany

**Keywords:** Coarctation of the aorta, Stents, Heart defects, Congenital, Avoidable costs, Cost savings

## Abstract

**Background:**

Undesirable outcomes in health care are associated with patient harm and substantial excess costs. Coarctation of the aorta (CoA), one of the most common congenital heart diseases, can be repaired with stenting but requires monitoring and subsequent interventions to detect and treat disease recurrence and aortic wall injuries. Avoidable costs associated with stenting in patients with CoA are unknown.

**Methods:**

We developed an economic model to calculate potentially avoidable costs in stenting treatment of CoA in the United Kingdom over 5 years. We calculated baseline costs for the intervention and potentially avoidable complications and follow-up interventions and compared these to the costs in hypothetical scenarios with improved treatment effectiveness and complication rates.

**Results:**

Baseline costs were £16 688 ($25 182) per patient. Avoidable costs ranged from £137 ($207) per patient in a scenario assuming a 10% reduction in aortic wall injuries and reinterventions at follow-up, to £1627 ($2455) in a Best-case scenario with 100% treatment success and no complications. Overall costs in the Best-case scenario were 90.2% of overall costs at Baseline. Reintervention rate at follow-up was identified as most influential lever for overall costs. Probabilistic sensitivity analysis showed a considerable degree of uncertainty for avoidable costs with widely overlapping 95% confidence intervals.

**Conclusions:**

Significant improvements in the treatment effectiveness and reductions in complication rates are required to realize discernible cost savings. Up to 10% of total baseline costs could be avoided in the best-case scenario. This highlights the need to pursue patient-specific treatment approaches which promise optimal outcomes.

**Electronic supplementary material:**

The online version of this article (doi:10.1186/s12913-017-2215-2) contains supplementary material, which is available to authorized users.

## Background

Despite a relatively low incidence of 96 per 10,000 live births [1], patients with congenital heart disease (CHD) consume a disproportionately large share of health care spending. Following often expensive primary surgery most patients can lead a normal life. However, the need for close monitoring and reinterventions at follow-up incur substantial costs. Hospital admissions among adults with CHD are 3.5 times more frequent than in the general population and at least half of all adult CHD patients is hospitalized over a 5-year period [2, 3]. Treatment complications further contribute to high resource use. An American multicentre study found that complications and extended hospital stay after surgery for CHD were associated with an average excess cost per case of $56 584 ($132 483 for major complications) [4]. Another study found complications to be associated with a 3-fold risk of high resource utilization (above the 90^th^ percentile) in CHD patients undergoing surgery [5].

Costs associated with undesirable outcomes, including treatment complications and reinterventions, can be quantified as avoidable costs. A clinical area where the economic impact of adverse effects can be studied is coarctation of the aorta (CoA), one of the most common CHD. CoA significantly reduces life expectancy and is associated with morbidity even after successful repair [6–8]. Implementation of a stent is an effective option to repair the obstructed site of the aorta, yet patients are still at risk of experiencing serious complications while undergoing treatment, and outcomes at follow-up show scope for significant improvement. Recurrent CoA and aortic wall injuries contribute to a reintervention rate after stenting of approximately 14% at follow-up [9].

The costs associated with undesirable outcomes including treatment-related complications of stenting among individuals with CoA are unknown. Our objective in this paper is to quantify the costs associated with such potentially avoidable outcomes.

## Methods

We developed an economic model to calculate the expected costs associated with stenting for CoA in the United Kingdom on a per patient basis. We included important costs of the initial intervention and monitoring, as well as costs of complications and follow-up interventions that are potentially amenable to improvements in stenting repair of CoA. We specified four hypothetical scenarios of improved treatment outcomes and compared the costs of each scenario to the baseline costs to obtain estimates of avoidable costs. Three scenarios were based on potential increases in stenting treatment success and reductions in complications and reintervention rates. The last scenario represents the ‘ideal’ scenario in which the probability of treatment success is 100% and no complications and reinterventions occur.

### Structure of the model

**Fig. 1:**
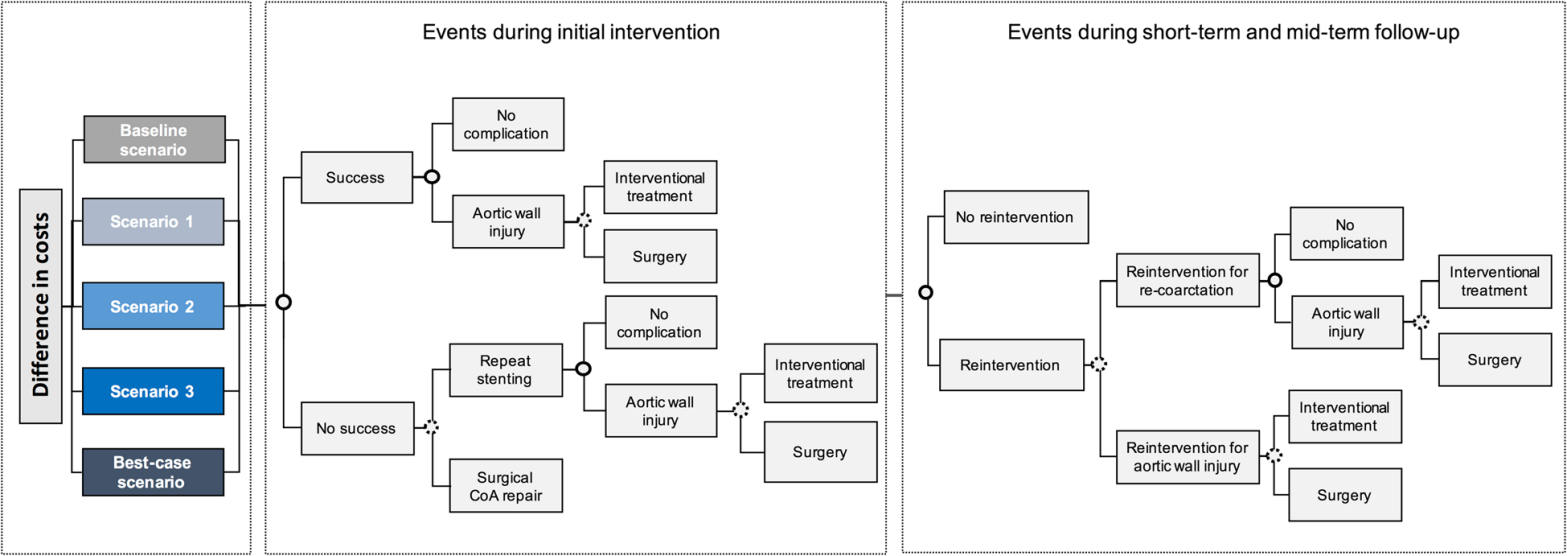
Analytical approach and model structure illustrating alternative events and their sequelae. The primary outcome of this analysis is difference in costs between the Baseline scenario and four hypothetical scenarios of improved treatment effectiveness and patient safety. All scenarios include the same events at initial intervention, short-term, and mid-term follow-up. Scenarios differ in the probabilities attached to events. The figure shows possible events in the first period of the model (during or immediately after the initial intervention), and at short- and mid-term follow-up. The same events are included for short- and mid-term follow-up. Hypertension medication and imaging are not dependent on any other events and are therefore not connected to the other events. Hypertension is not directly influenced by other events at follow-up and does not impact on complications or reintervention rates itself. Imaging is recommended for all patients after CoA repair at least every two years [10]. Full circles indicate event probabilities subject to change in scenarios. Dotted circles indicate exogenous event probabilities

The analytical approach of comparing expected costs in the Baseline scenario, Scenarios 1–3, and Best-case scenario, is presented in Fig. 1. The model structure of events and sequelae remained constant for all scenarios, with varying probabilities for events subject to change in the scenarios.

Figure 1 depicts the model during initial intervention and at follow-up. Since all patients undergo stenting, the model consists exclusively of chance nodes and branches emanating from this treatment decision. Each chance node has a set of mutually exclusive events which are displayed in the model structure as boxes. Each event is associated with costs. The value of each chance node is determined by the probabilities and costs attached to its events.

The first chance node in the model is success at the initial stenting intervention. Stenting success is defined as achieving a post-treatment blood pressure gradient ≤20 mmHg, which represents the threshold for intervention indication in patients with CoA [10, 11]. Aortic wall injuries caused by the catheter or dilatation of the stenotic aortic segment are possible despite successful gradient reduction, as indicated by the next chance node. In this model, we only included aortic wall injuries that warrant intervention. In some cases, aneurysms will develop over the years following the intervention. In order to assign a cost to these complications, our model includes them at the time when they require treatment, i.e. either immediately after the initial intervention, or at short- or mid-term follow-up. The branch of aortic wall injuries leads to a chance node where repair of the injury is conducted either percutaneously or surgically.

In the lower branch of the initial intervention period model, we assumed that patients who did not achieve a post-intervention treatment gradient ≤20 mmHg underwent a second intervention, which could be either repeat stenting or surgery. The branch for repeat stenting then leads to a chance node of experiencing an aortic wall injury, and further to the percutaneous or surgical treatment of the injury.

The model includes the same events for the short-term and mid-term follow-up period, although different probabilities are assigned to them according to the specific period. The branches emanating from the first chance node at follow-up are either no reintervention or reintervention due to aortic wall injuries or recurrence of coarctation. The branch for reintervention for re-coarctation is identical to that after successful stenting at the initial intervention. The branch for reintervention due to aortic wall injury leads to a chance node of percutaneous or surgical treatment.

We applied a 5-year timeframe for our analysis, distinguishing between costs and events during or immediately after the initial treatment; during short-term follow-up (3–18 months); and mid-term follow-up (18 months–5 years). Expected costs were obtained for each of these three time periods separately. Discounting the results at short- and mid-term follow-up, we also calculated expected overall costs over a 5-year period. The time frame was chosen due to limited availability of reliable data beyond 5 years of follow-up.

Actuarial survival of non-infant patients after CoA stenting repair is almost 100% even 10 years after treatment [12]. We assumed no mortality in the model period. Since there is no loss to mortality in the transition between initial intervention, short-term, and mid-term follow-up, all three time periods can be combined to give the overall expected costs of the treatment over a course of 5 years.

#### Model input

Baseline values for event probabilities and costs are presented in Tables 1 and 2, respectively.

**Table 1:** Event probabilities

	Probability estimate	SD	Distribution	Source
Event				
Treatment success after stenting	0.967	0.0128	Beta	[9]
Intervention after unsuccessful stenting:				
Repeat stenting	0.5	0.1	Beta	Assumption
Surgery	0.5	0.1	Beta	Assumption, inverse probability of repeat stenting
Aortic wall injury after stenting	0.008	0.0026	Beta	[12, 28–40]
Intervention after aortic wall injury:				
Percutaneous treatment	0.9	0.02	Beta	Assumption
Surgery	0.1	0.02	Beta	Assumption, inverse probability of percutaneous treatment
Short-term follow-up				
Imaging	1	0.01	Beta	[10]
Patients requiring anti-hypertension medication	0.43	0.0301	Beta	[31, 32]
Patients requiring reintervention	0.091	0.0286	Beta	[30, 32]
Reintervention for aortic wall injury	0.1	0.02	Beta	Assumption, based on literature [30–32, 40]
Reintervention for re-coarctation	0.9	0.02	Beta	Assumption, inverse probability of reintervention for aortic wall injury
Mid-term follow-up				
Imaging	1	0.01	Beta	[10]
Patients requiring anti-hypertension medication	0.389	0.1051	Beta	[28, 31, 32, 38]
Patients requiring reintervention	0.185	0.0413	Beta	[12, 28, 29, 31, 33, 34, 36, 38, 39]
Reintervention for aortic wall injury	0.5	0.02	Beta	Assumption, based on literature [12, 28, 29, 31–34, 36, 38, 39]
Reintervention for re-coarctation	0.95	0.02	Beta	Assumption, inverse probability of reintervention for aortic wall injury

**Table 2:** Cost estimates

Item	Estimate (£)	SD	Distribution	Source	Comment
Stenting for CoA	£4507.72	£2612.84	Gamma	[41]	Currency codes YR12Z; YR13Z; YR14A; YR14B; YR15A; YR15B; YR15C
Surgery for CoA	£7497.61	£5600.01	Gamma	[41]	Currency codes EC01A; EC01B; EC01C; EC02A; EC02B; EC02C; EC03A; EC03B; EC03C
Aortic wall injury requiring interventional treatment	£10 913.59	£5790.90	Gamma	[41]	Currency codes YR01Z; YR02Z; YR20Z
Aortic wall injury requiring surgery	£8545.20	£2305.29	Gamma	[41]	Currency codes YQ01A; YQ01B; YQ02Z; YQ03A; YQ03B
Follow-up imaging	£5660.04	£1698.01	Gamma	[41]	Currency code YZ04Z
Hypertension medication	£67.53	£20.26	Gamma	[42]	Average cost per patient per year. Medication plus annual check-up with GP. Adjusted to 2014 prices.

### Event probabilities

Estimates of event probabilities were derived primarily from a meta-analysis comparing the effectiveness of stenting and balloon dilatation in CoA patients [9]. For event probabilities at different follow-up periods, we used pooled estimates for aortic wall injuries after stenting; overall reintervention rates at short- and mid-term follow-up; and the proportion of patients requiring anti-hypertensive medication at short- and mid-term follow-up. Our data were based on studies that were randomized controlled trials with a minimum of 50 participants, or other study designs with at least 50 participants and that were conducted in high income countries. Additional details are available elsewhere [9].

We made assumptions about the proportion of patients undergoing repeat stenting (as opposed to surgery) after unsuccessful initial stenting repair; the proportion of patients receiving percutaneous treatment (as opposed to surgery) after aortic wall injury; and the proportion of patients requiring reintervention due to aortic wall injury (as opposed to recurrent CoA) in both short-term and mid-term follow-up periods of the model. Sensitivity of our findings to assumptions was extensively tested, as described below.

### Cost estimates

Our model adopted the health care provider’s perspective and included direct costs only.

Cost estimates for the treatment of CoA and complications arising from the initial treatment are based primarily on United Kingdom National Health Service (NHS) reference costs 2013-14. The NHS provides the average unit treatment costs for every fiscal year, collected from all NHS trusts. Reference costs reflect the full costs of providing services [13]. NHS reference costs do not distinguish between reasons for admissions. To obtain estimates for cost input parameters we first identified all relevant variants of treatments (currency codes). For each cost item in our model we then calculated average costs from the currency codes relating to that item. We weighted average costs with the number of healthcare resource groups (HRG) performed annually. For example, we obtained an estimate of the cost of stenting for CoA by adding up all treatments with stents in peripheral arteries (non-coronary stents) and subsequently weighting them based on their frequency. Our cost estimates are likely to be conservative as they are based on all patients undergoing any given intervention instead of exclusively CHD patients who are typically complex cases with multiple comorbidities.

All costs were discounted at the standard NHS discount rate of 3.5% per year. Where necessary we adjusted costs to 2014 Great British Pounds. We assumed health care costs to increase 2 percentage points above annual inflation rate [14].

#### Analytic strategy

The primary outcome of our analysis was expected avoidable cost associated with stenting in patients with CoA. In our base case analysis, we calculated the expected costs for the immediate treatment period, expected costs at short-term follow-up, and expected costs at mid-term follow-up by multiplying the event probabilities with their associated costs in each branch of the model. We then summed the expected costs from the three periods to obtain an estimate of the total expected costs. We discounted the expected costs at short-term and mid-term follow-up at 3.5% p.a.

**Fig. 2:**
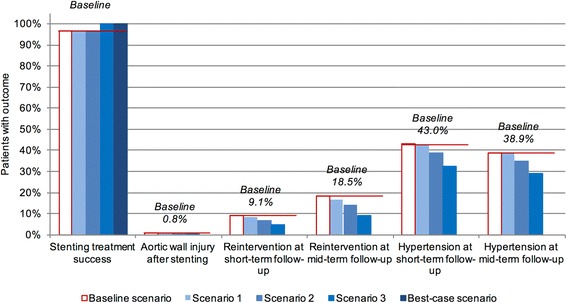
Input probabilities for scenarios. Figure shows probabilities of events subject to change in the four scenarios compared to the Baseline scenario (red line). No bar shown for Best-case scenario for aortic wall injury; reintervention; and hypertension because the probability for these events is 0.0% in this scenario

We compared the costs of this Baseline scenario, including costs incurred by complications and other undesired events, with the costs that would incur if no or fewer undesired events occurred [15, 16]. We refer to the difference between the baseline cost estimate and the cost estimate in such scenario as the avoidable costs associated with stenting for CoA. In addition to the Baseline scenario, we specified four scenarios with varying rates of hypothetical treatment success and rates of complications (Fig. 2). We calculated expected avoidable costs compared to the Baseline scenario for each of the four scenarios.

### Scenario 1

In the first scenario, the proportion of patients with initial treatment success remains constant at the baseline value of 96.7%. Proportion of patients with aortic wall injuries decreases by 10% to 0.7%. Proportion of patients with reinterventions at follow-up decreases by 10% to 8.2% at short-term and 16.7% at mid-term follow-up. Hypertension medication is required by the baseline proportion of 43.0% of patients at short-term and 38.9% at mid-term follow-up.

### Scenario 2

Scenario 2 assumes a constant initial treatment success rate. Both aortic wall injuries and follow-up reinterventions are assumed to be reduced by 25% from baseline. Proportion of patients with aortic wall injuries is 0.6% in this scenario. Reintervention rates at short-term and mid-term follow-up are 6.8% and 13.9%, respectively. Furthermore, a 10% reduction in the proportion of patients requiring anti-hypertensive medication to 38.7% at short-term and 35.0% at mid-term follow-up is assumed.

### Scenario 3

In the third scenario, the initial treatment success rate increases to 100%. Aortic wall injuries are reduced by 50% from baseline to a proportion of 0.4% of patients. Reinterventions at follow-up are also reduced by 50% from baseline, leading to values of 4.6% at short-term and 9.3% at mid-term follow-up. The proportion of patients requiring anti-hypertensive medication is assumed to be reduced by 25% to 32.3% at short-term and 29.2% at mid-term follow-up.

### Best-case scenario

Finally, we calculate the costs of stenting for CoA under ideal conditions. In this scenario, treatment success is 100%, with no complications during the initial treatment and at follow-up. The scenario assumes that no reinterventions are necessary and that no patient requires anti-hypertensive medication.

#### Sensitivity analysis

##### Deterministic sensitivity analysis

We conducted one-way sensitivity analyses. We varied key probability and cost inputs by 10% and plotted the effect on costs avoided compared to baseline, in a tornado diagram. The tornado diagram presents the relative impact that each input factor has on the outcome, holding all other variables constant.

We then varied input variables for all five scenarios to assess the sensitivity of the model with respect to (1) the overall level of input values, and (2) the expected avoidable costs in the scenarios relative to the Baseline scenario. Holding all other parameters constant, we inspected the extent to which each parameter had an impact on the difference in avoidable costs in the four scenarios. We did not include the Best-case scenario in one-way sensitivity analyses of parameters that were assumed to be ideal (i.e. 100% treatment success and 0% complication rates) in this scenario.

We chose a range from 0 to twice the initial input value for event probabilities and half to twice the initial value for costs in most cases.

##### Probabilistic sensitivity analysis

We conducted probabilistic sensitivity analysis (PSA) to simultaneously take into account the uncertainty associated with all input factors in our model.

We generated 1000 random values for each input parameter based on its point estimate and standard deviation (SD). We assigned a beta distribution to event probabilities and a gamma distribution to cost estimates. The values were therefore restricted to between 0 and 1 for probabilities, and to non-negative values for costs. SD for cost estimates were derived from published NHS reference costs for procedures at the aggregate level, which did not show much variation, potentially underestimating SD. We assessed the impact of potentially underestimated SD of cost parameters on our primary outcome, avoidable costs, by rerunning PSA with inflated SD (twice the initial value).

We then ran 1000 iterations of the model. Each iteration used a different value of the random value distribution of input parameters. We summarized the results of PSA using the mean value of the 1000 iterations and its 95% confidence interval (CI). We also present all 1000 PSA estimates of total costs for each of the scenarios (including Baseline) in a single diagram, which demonstrates the uncertainty surrounding estimates of total costs in the model.

The model and sensitivity analyses were implemented in Microsoft Excel (Microsoft; Redmond, WA).

## Results

### Results of base-case analysis

Expected costs per patient at initial treatment, short-term, and mid-term, as well as overall expected costs are displayed for Baseline, Scenarios 1 to 3, and Best-case scenario in Table 3. The last row shows expected avoidable costs compared to Baseline for all four scenarios.

**Table 3:** Results from base-case analysis: expected costs of stenting for CoA and avoidable costs in four scenarios

	Baseline	Scenario 1	Scenario 2	Scenario 3	Best-case scenario
Expected costs initial treatment	£4790	£4781	£4769	£4550	£4508
Expected costs short-term	£5980	£5933	£5861	£5741	£5492
Expected costs mid-term	£5919	£5836	£5709	£5498	£5061
Expected costs overall	£16,688	£16,551	£16,338	£15,790	£15,061
Expected avoidable costs vs. Baseline		£137	£350	£898	£1627

We calculated the overall expected costs per patient as £16 688 ($25 181; exchange rate per December 4, 2015). Expected overall costs in Scenario 1 were £16 551 ($24 975) with avoidable costs amounting to £137 ($207).

Scenario 2 showed slightly lower overall costs (£16 338/$24 654) and conversely higher savings from avoidable costs (£350/$528). Optimistic assumptions in Scenario 3, including a 100% initial treatment success rate, brought overall expected costs down to £15 790 ($23 827) with expected avoidable costs at an estimated £898 ($1355).

In the Best-case scenario, overall expected costs were £15 061 ($22 727), translating into £1627 ($2455) in avoidable costs. The most substantial cost savings compared to Baseline were accumulated at follow-up (£488/$736 at short-term and £858/$1295 at mid-term), while avoidable costs at the initial treatment amounted to £282 ($426).

### Deterministic sensitivity analysis

**Fig. 3:**
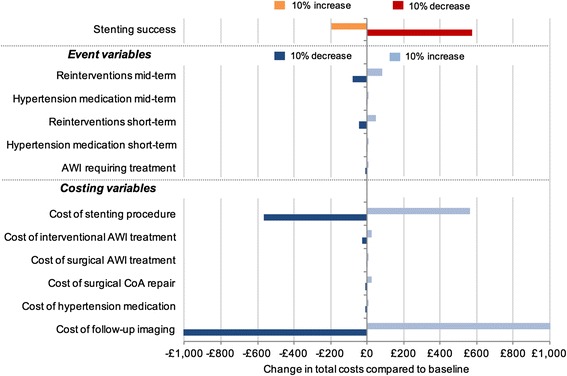
Tornado diagram

The tornado diagram (Fig. 3) shows that cost of imaging at follow-up; cost of the stenting procedure; and probability of successful stenting have the largest impact on total costs, when considering a 10% increase or decrease in key model parameters while holding all other parameters constant.

We assessed the sensitivity of expected avoidable costs in the four scenarios with respect to all model input parameters. We only display results of univariate analysis for stenting treatment success and reintervention rates. Sensitivity analyses of all parameters are available in the Additional file 1 (section A3).

**Fig. 4:**
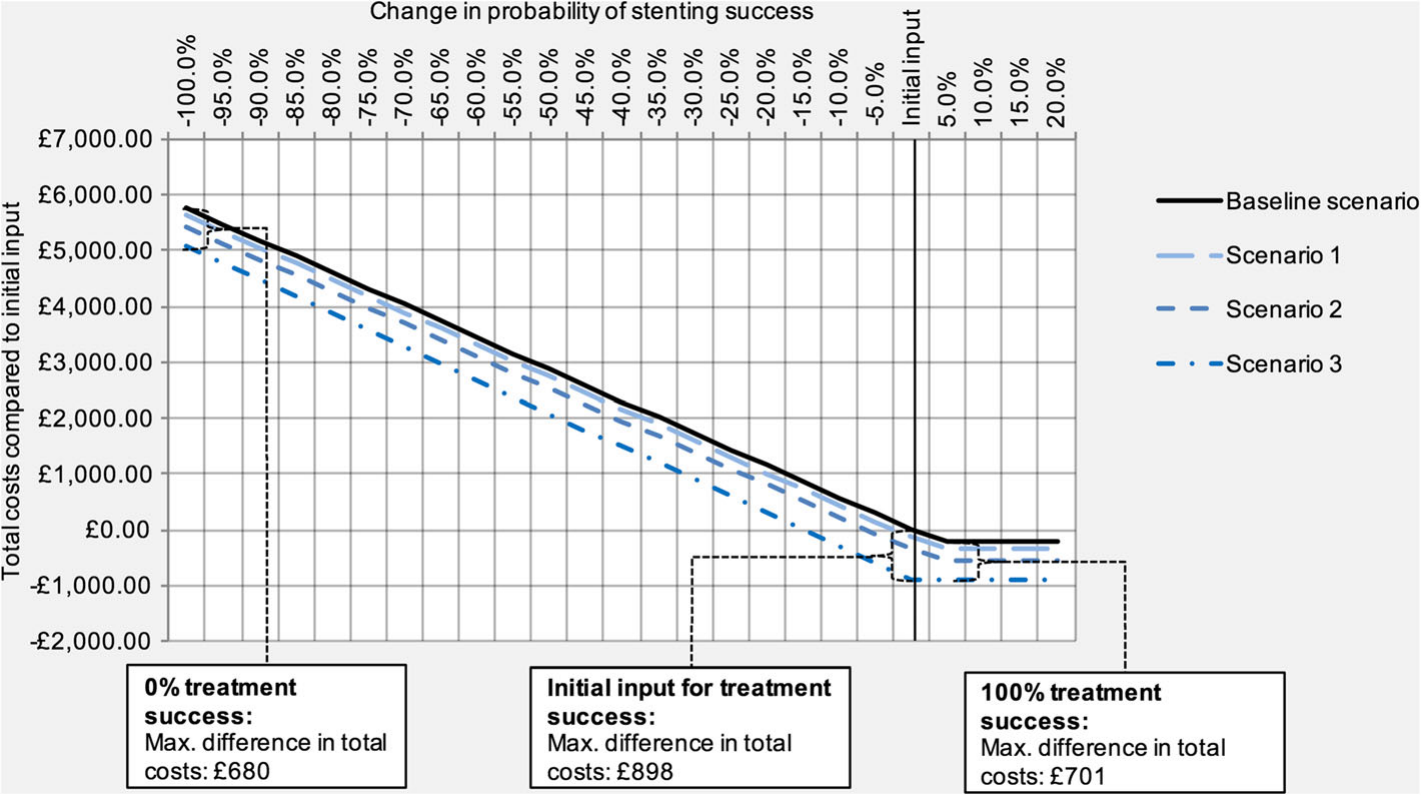
Univariate sensitivity analysis for stenting success. The diagram shows the relationship between varying probabilities for treatment success (horizontal axis) and expected total costs compared to the initial input value (vertical axis) in Scenarios 1–3, as well as the Baseline scenario. Varying values of treatment success are displayed relative to the initial input (96.7% treatment success)

Figure 4 displays expected avoidable costs in Scenarios 1–3 and the Baseline scenario when varying treatment success rate from a decrease by 100% from the initial value to an increase by 20%. At the initial input level there were no expected avoidable costs in the Baseline scenario and the maximum avoidable costs were seen in Scenario 3 at £898 (Best-case scenario, at a constant success rate of 100%, was not included in the sensitivity analysis). Even when varying treatment success rates to the extreme values of 0 and 100%, the results for maximum avoidable costs did not differ substantially from that obtained with the initial input value. At a rate of 0% treatment success, total costs in all four scenarios were considerably higher. The difference in total costs, however, decreased only slightly (from £898 to £680 between Baseline and Scenario 3). The estimates for all four scenarios reached a floor at treatment success improvement rates over 3.5%, as this coincided with achieving treatment success in 100% of patients.

**Fig. 5:**
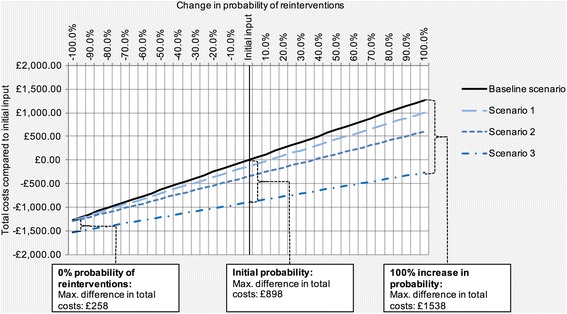
Univariate sensitivity analysis for follow-up reinterventions. The diagram shows the relationship between varying probabilities for reinterventions at follow-up (horizontal axis) and expected total costs compared to the initial input value (vertical axis) in Scenarios 1–3, as well as the Baseline scenario. Varying values of reintervention rates are displayed relative to the initial input of 9.1% at short-term and 18.5% at mid-term follow-up

At the extremes of the sensitivity analysis for reinterventions at follow-up, we observed significant differences between the magnitude of avoidable costs associated with the four scenarios (Fig. 5). Compared to avoidable costs of £898 in Scenario 3 associated with the initial parameter values, the difference in total costs between the Baseline scenario and Scenario 3 was £258 when 0% of patients required reinterventions at follow-up, and increased to £1538 when the estimate was set to twice its initial value.

### Probabilistic sensitivity analysis

**Fig. 6:**
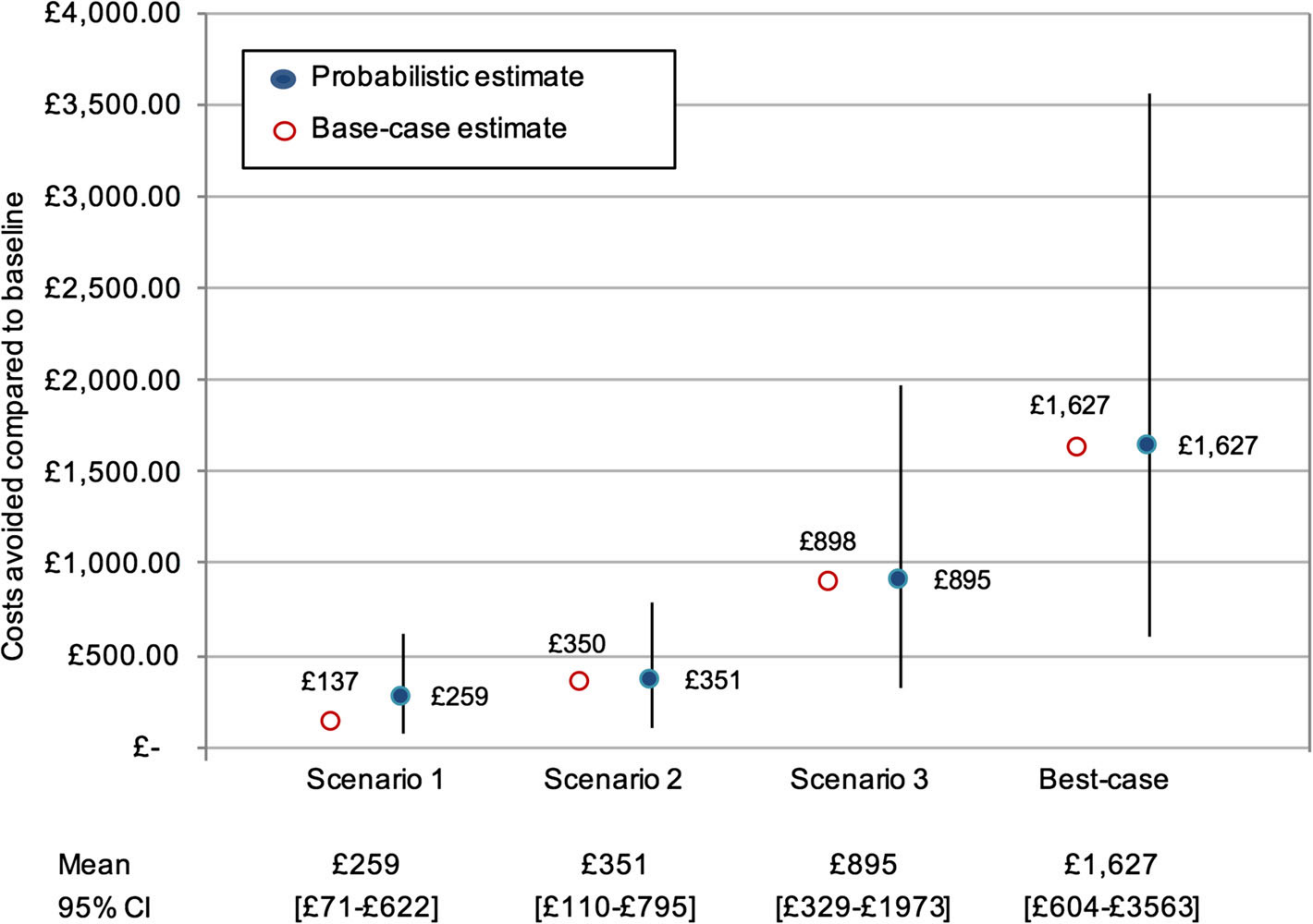
Estimated avoidable costs in four scenarios; probabilistic sensitivity analysis. Blue circles represent mean expected avoidable costs compared to Baseline, with bars indicating 95% CIs. Red circles show estimates of the base-case analysis

After 1000 iterations of the model using random values drawn from relevant distributions for each input parameter, mean estimated avoidable costs were similar to expected avoidable costs in the deterministic base case analysis (Fig. 6). This is particularly true for Scenarios 2, 3, and Best-case, where only small deviations occurred in point estimates between base case analysis and PSA. Avoidable costs in Scenario 1 were estimated at £259 (95% CI 71-622) in PSA compared to £137 in the base case analysis.

The results of PSA show a wide overlap of 95% CIs between avoidable costs in Scenarios 1 and 2, and to some extent between Scenarios 2 and 3. There was minimal overlap in the 95% CIs between Scenario 1 and Best-case (full results of PSA for all scenarios shown in Additional file 1, section A1).

**Fig. 7:**
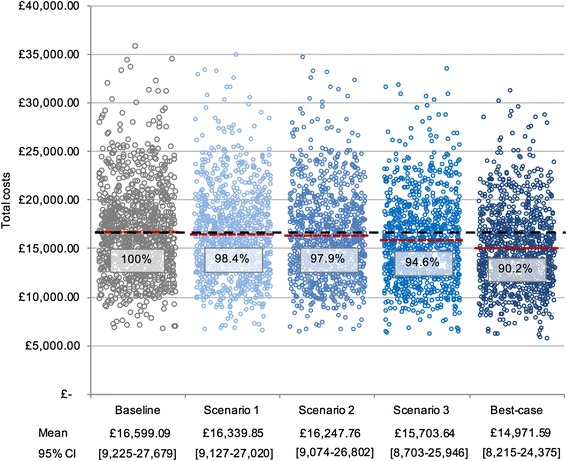
Estimated total costs; probabilistic sensitivity analysis. Results of PSA of total costs at Baseline and in four scenarios. Each circle represents expected total costs in one of *n* = 1000 iterations. Red bars show the mean result of iterations

PSA of total costs showed wide overlap between the Baseline scenario and all four scenarios (Fig. 7). Total costs in the Best-case scenario were 90.2% of costs in the Baseline scenario. Potential cost savings in the other scenarios ranged from 1.6 to 5.4% of total costs at baseline.

Sensitivity analysis of the SD of cost parameters did not reveal a significant impact on our main results. After inflating SD of all cost parameters to twice their initial value mean expected avoidable costs in the four scenarios did not deviate from the results obtained with unadjusted SD (results of SD sensitivity analysis shown in Additional file 1, section A2).

## Discussion

Proposals for cost reduction in health care are often met with skepticism. Cost cuts are often perceived to be associated with lower staff-per-patient ratios, reduced investment and, consequently, worse quality in care provision. However, high treatment costs are not necessarily a reliable indicator of high quality [17]. There is an area where cost reductions are unlikely to meet any resistance: sub-optimal provision of care and complications associated with treatments are both harmful (for patients) and costly. Berwick and colleagues estimated the annual cost of failures of care delivery in the US health care system between $102 and $154 billion [18]. Costs associated with theoretically avoidable complications and potential room for improvement in effectiveness can be labelled as avoidable costs and provide an attractive target for cost reduction efforts. Simultaneous cost savings and improvement of patient outcomes have been demonstrated in various settings [19–22].

To the best of our knowledge, this analysis of avoidable costs in stenting therapy for patients with CoA is the first to examine the potential for cost savings through increased treatment effectiveness in CHD.

We found that significant improvements in treatment effectiveness are required to achieve discernible cost savings. Initial treatment success of stenting in patients with CoA is already very high, limiting the scope for improvements in treatment effectiveness and accompanying cost reductions from averted repeat procedures. The tornado diagram suggested that cost of imaging at follow-up; cost of the stenting procedure; and probability of successful stenting had the largest impact on total costs. This is not surprising given that all patients in the model undergo both stenting and imaging at follow-up. Varying values for the cost of the stenting procedure and follow-up imaging impact on the overall cost levels, but possess little influence on avoidable costs as defined in our analysis. Our analysis identified reinterventions at follow-up as the most important lever for achieving cost savings. Aneurysm formation and recurrent CoA, along with hypertension, have long been identified as weaknesses of CoA repair [23]. We observe that reinterventions due to aortic wall injuries and recurrent CoA are also the main drivers of avoidable costs in stenting repair of CoA.

If discernible savings through the reduction of potentially avoidable adverse outcomes are to be realized, clinical practice has to approach the best-case scenario. Personalized treatment decisions through the use of individual patient data are a promising path towards treatment optimization. Stenting is an accepted treatment option for CoA but details of the intervention, including choice of stent type and length, as well as timing of the intervention, are likely to be best decided for each patient individually. Treatment decisions must take into account the patient’s age, anatomy, treatment history, and lifestyle. Recent advances in the development of Virtual Physiological Human (VPH) projects have the potential to move clinical practice towards increasingly individualized and patient-centered treatment decisions with better patient outcomes. Just as in other areas of personalized medicine, potential benefits are manifold and include improving mortality and morbidity, and reducing health care expenditures. In patients with CoA, image-based modelling of the aorta was recently used to create a virtual stenting tool which could aid clinical decision-making by simulating post-treatment blood flows in various scenarios [24, 25]. Given the overall good performance of stenting repair of CoA, cost effectiveness of this approach will be conditional on improved identification of selected, high-risk populations for whom tailored interventions can prevent future high-cost complications and treatments.

Overall, the overall scope for cost savings from improved treatment outcomes in patients undergoing stenting for CoA is small. Cost savings per patient were £137, £350, and £898 in Scenarios 1, 2, and 3, respectively. In the United States, an estimated 521 stenting procedures are conducted annually in patients with CoA [26]. Under the assumptions that prevalence of CoA is similar in the United Kingdom and the United States, and that procedure frequency is relative to total population, a back of the envelope calculation reveals cost savings for the NHS of £14 522 in Scenario 1, £37 100 in Scenario 2, and £95 188 in Scenario 3 over 5 years for 106 patients yearly.

### Limitations

Our analysis was limited by the data available to model undesirable outcomes at follow-up. We included reinterventions due to aortic wall injuries and recurrent CoA as well as antihypertensive medication therapy but did not account for other complications that might occur late after CoA repair due to limited data availability. Similarly, it was not always possible to include estimates of event probabilities from meta-analyses or large trials due to limited availability of such studies and some of the parameters of our model were therefore based on assumptions informed by reports in the literature. Our model was also restricted to a 5-year period. Long-term follow-up studies of patients undergoing stenting are still relatively rare and often conducted retrospectively in small cohorts. For example, long-term studies are required to assess the consequences of exercise-induced hypertension after CoA repair [27].

It is unclear through which mechanisms the improvements in the quality of care for patients with CoA included in our scenario analysis would be achieved and whether such improvements are realistic. For example, whether or not CoA repair can resolve hypertension in patients with CoA remains elusive. Perfect outcomes in all patients, as modeled the Best-case scenario, might never be accomplished. However, our study was designed not only to demonstrate what could realistically be achieved, but also to demonstrate the absolute maximum in cost savings associated with stenting repair of CoA.

We focused on costs and events that were related to stenting repair of CoA. We did not aim to obtain estimates of all costs and events associated with CoA. Expected costs of stenting for CoA repair therefore do not represent a true estimate of all costs associated with the disease.

Cost estimates were restricted to aggregate level information from the United Kingdom NHS reference costs. It is possible that mean costs of procedures are higher for patients with CHD than other patients due to comorbidities. A potential systematic underestimation of all cost parameters included in the model has implications for the overall cost level but should not affect relative comparisons of avoidable costs in the specified scenarios. PSA results demonstrated a considerable degree of uncertainty associated with the results of the model, which stems from relatively small potential cost savings compared to total costs as well as from uncertainty in input parameters. Total costs in the four scenarios ranged from 98.5 to 90.2% of Baseline costs.

We derived SD for cost estimates from aggregate cost items in the NHS reference costs. It is therefore possible that SD were underestimated for the procedures included in the model. Apart from the expected increase in variance, inflating SD to twice their initial value did not change our results.

### Conclusion

We examined the potential cost savings associated with improvement in effectiveness and reduction of complications in stenting treatment of aortic coarctation. The biggest potential for cost savings lies in improving the endurance of therapies, and thus the reduction of reintervention rates at follow-up. Significant improvements in the treatment effectiveness and reductions in complication rates are required to realize discernible cost savings. Up to 10% of total baseline costs could be avoided in the best-case scenario. This highlights the need to pursue patient-specific treatment approaches which promise optimal outcomes. The individualization of treatment procedures with the help of disease specific modeling can already help to select the most relevant stenosis and to find an optimal location for interventional treatment. Further advances in modelling will potentially enable physicians to identify patients with high risk of treatment failure and prevent costly complications and follow-up treatment in those.

## Additional file

Additional file 1: The supplemental material provides additional information on model inputs and results of the extensive sensitivity analyses conducted. The supplemental material is provided in one document under the following section headings: A1. Results of probabilistic sensitivity analysis. A2. Calculation of cost inputs and standard deviations. A3. Univariate sensitivity analyses of all input parameters. (PDF 1.14 mb)

## Abbreviations

CHD: Congenital heart disease
CI: Confidence interval
CoA: Coarctation of the aorta
NHS: National Health Service
PSA: Probabilistic sensitivity analysis
SD: Standard deviation
