# Supplementary material for: Avoidable costs of stenting for aortic coarctation in the United Kingdom: an economic model
Source: BMC Health Serv Res. 2017 Apr 10;17:258. doi: 10.1186/s12913-017-2215-2 (PMC5387244; doi:10.1186/s12913-017-2215-2)
Supplement: Additional file 1: — The supplemental material provides additional information on model inputs and results of the extensive sensitivity analyses conducted. The supplemental material is provided in one document under the following section headings: A1. Results of probabilistic sensitivity analysis. A2. Calculation of cost inputs and standard deviations. A3. Univariate sensitivity analyses of all input parameters. (PDF 1.14 mb) [file 12913_2017_2215_MOESM1_ESM.pdf]

ADDITIONAL FILE

Supplemental material for

AVOIDABLE COSTS OF STENTING FOR AORTIC COARCTATION IN THE UNITED KINGDOM: AN ECONOMIC MODEL

Maximilian Salcher MSc[1*]; Alistair Mcguire PhD[1]; Vivek Muthurangu MD[2]; Marcus Kelm MD[3]; Titus Kuehne MD[3]; Huseyin Naci PhD[1]

[1] LSE Health, London School of Economics and Political Science, London, United Kingdom

[2] UCL Institute of Cardiovascular Science & Great Ormond Street Hospital for Children, Great Ormond Street Hospital, London, United Kingdom

[3] Department of Paediatric Cardiology and Congenital Heart Diseases, Deutsches Herzzentrum Berlin, Berlin, Germany

* Corresponding Author.

Address for correspondence: LSE Health and Social Care, Cowdray House; London School of Economics and Political Science; Houghton Street, London WC2A 2AE, United Kingdom.
Tel: +44 2079556959
Email: m.salcher@lse.ac.uk

Contents

A1. Results of probabilistic sensitivity analysis 2
A2. Calculation of cost inputs and standard deviations 3
A2.1 Effect of inflated standard deviations on expected avoidable costs 3
A3. Univariate sensitivity analyses of all input parameters 4

A1. Results of probabilistic sensitivity analysis

Table A 1: Results from probabilistic sensitivity analysis: expected costs of stenting for CoA and avoidable costs in four scenarios

	Baseline	Scenario 1	Scenario 2	Scenario 3	Best Case
Expected costs initial treatment	£4817 [1039-11 776]	£4809 [1034-11 762]	£4796 [1027-11 762]	£4583 [889-11 328]	£4540 [825-11 299]
Expected costs short-term	£5921 [3089-9 509]	£5874 [3049-9455]	£5801 [2981-9332]	£5680 [2879-9223]	£5429 [2639-9007]
Expected costs mid-term	£5861 [3232-9 342]	£5657 [3070-9068]	£5651 [3064-9062]	£5440 [2886-8775]	£5003 [2432-8300]
**Expected costs overall**	**£16 599 [9225-27 679]**	**£16 340 [9127-27 020]**	**£16 248 [9074-26 802]**	**£15 704 [8703-25 946]**	**£14 972 [8215-24 375]**
**Expected costs avoided vs. baseline**		**£259 [71-622]**	**£351 [110-795]**	**£895 [329-1973]**	**£1628 [604-3563]**

A2. Calculation of cost inputs and standard deviations

We obtained cost parameters from NHS reference costs spell level data (2013-2014). Costs reported in the NHS reference costs are average costs of procedures, without any indication of the range of costs individual patients incur.

For each item (*I*) included in our model we extracted the costs (*C*) of all relevant procedures (*P*) from the United Kingdom NHS reference costs and the number of patients (*N*) who had undergone them in 2013-2014. We calculated the average cost of *I* by adding up all *P*s, weighted with *N*. We obtained the standard deviation (SD) of the average cost from the variance between costs of individual procedures. Given that they originate from a sample of average costs of all patients undergoing a set of procedures it is possible that SD for items in our model are underestimated. We therefore inspected whether larger SD would influence our results.

A2.1 Effect of inflated standard deviations on expected avoidable costs

Figure A 1 shows point estimates and 95% confidence intervals of probabilistic estimates of avoidable costs in the four scenarios. As expected, estimates using inflated SD have larger variance. However, point estimates are very close to those obtained with unadjusted SD.

Figure A 1

Figure shows probabilistic estimates for avoidable costs in four scenarios compared to Baseline. Light blue circles represent estimates from analysis with unadjusted SD for cost parameters and dark blue circles estimates from analysis with inflated SD (2x initial value). Bars indicate 95% CI.

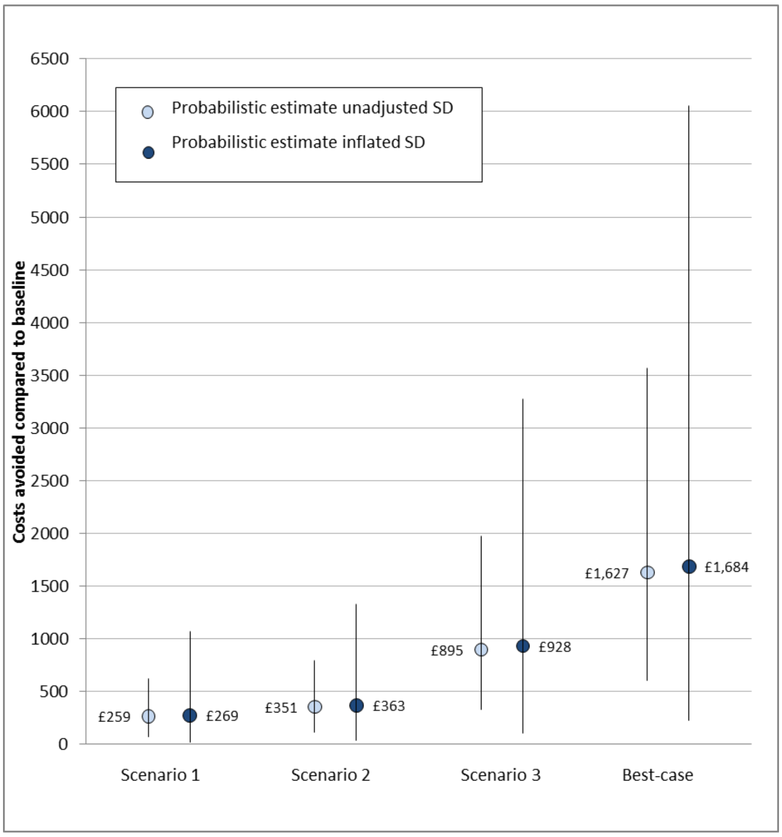

A3. Univariate sensitivity analyses of all input parameters

Below we present one-way sensitivity analysis graphs for all model input parameters. Cost inputs are presented in Figures A2-A7 and event probabilities in Figures A8-A14.

Figure A 2: Sensitivity analysis for cost of stenting

The diagram shows the relationship between varying costs for stenting (horizontal axis) and expected total costs compared to the initial stenting cost (vertical axis) in each of the four scenarios, as well as the baseline scenario. Varying values of stenting cost are displayed relative to the initial input of £5408.

The range of plausible cost estimates includes values reported for stenting and surgery in CoA patients from studies conducted in Sweden (purchasing power parity adjusted 2014-£'s 7913) [1] and the United States (PPP 2014-£'s7596) [2].

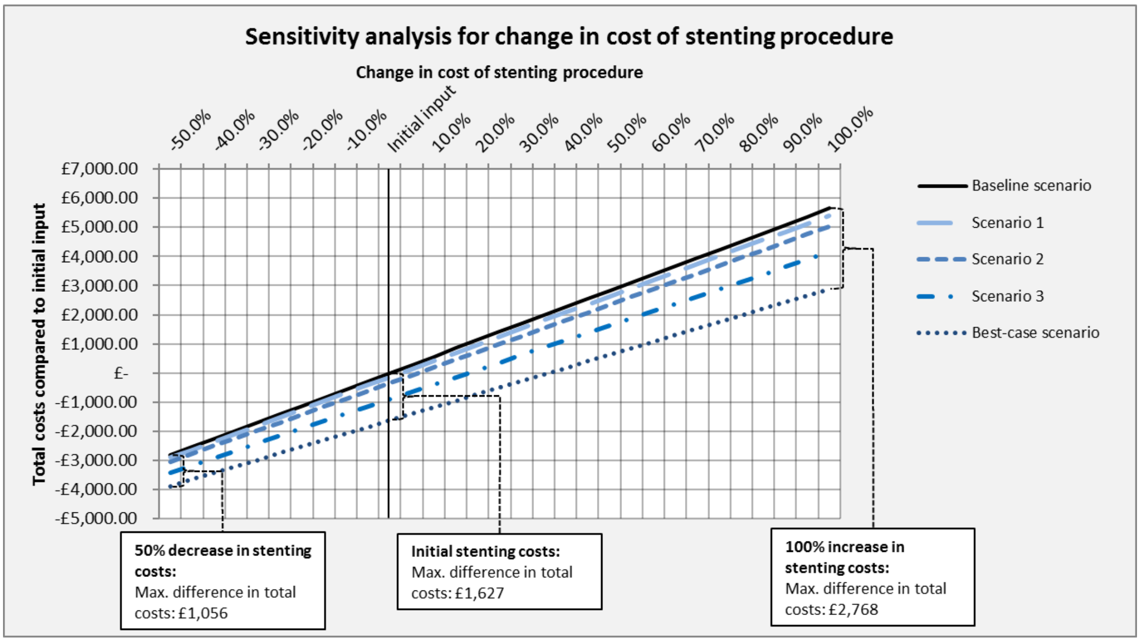

Figure A 3: Sensitivity analysis for cost of interventional aortic wall injury treatment

The diagram shows the relationship between varying costs for interventional aortic wall injury treatment (horizontal axis) and expected total costs compared to the initial treatment cost (vertical axis) in each of the four scenarios, as well as the baseline scenario. Varying values of treatment cost are displayed relative to the initial input of £10 914.

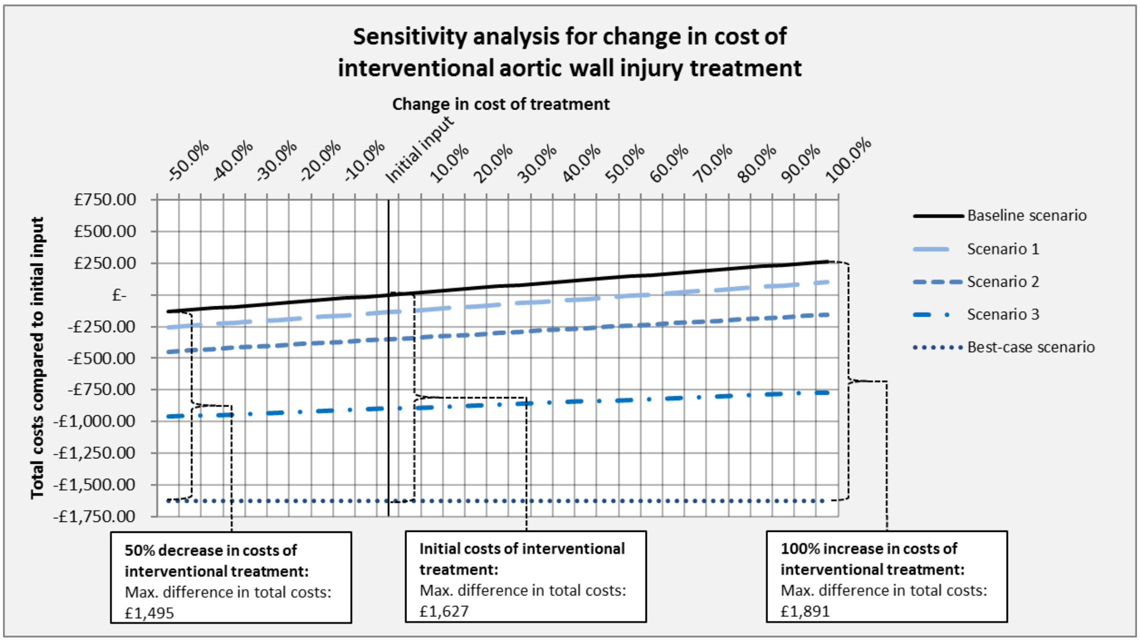

Figure A 4: Sensitivity analysis for cost of surgical aortic wall injury treatment

The diagram shows the relationship between varying costs for surgical aortic wall injury treatment (horizontal axis) and expected total costs compared to the initial treatment cost (vertical axis) in each of the four scenarios, as well as the baseline scenario. Varying values of surgical cost are displayed relative to the initial input of £8545.

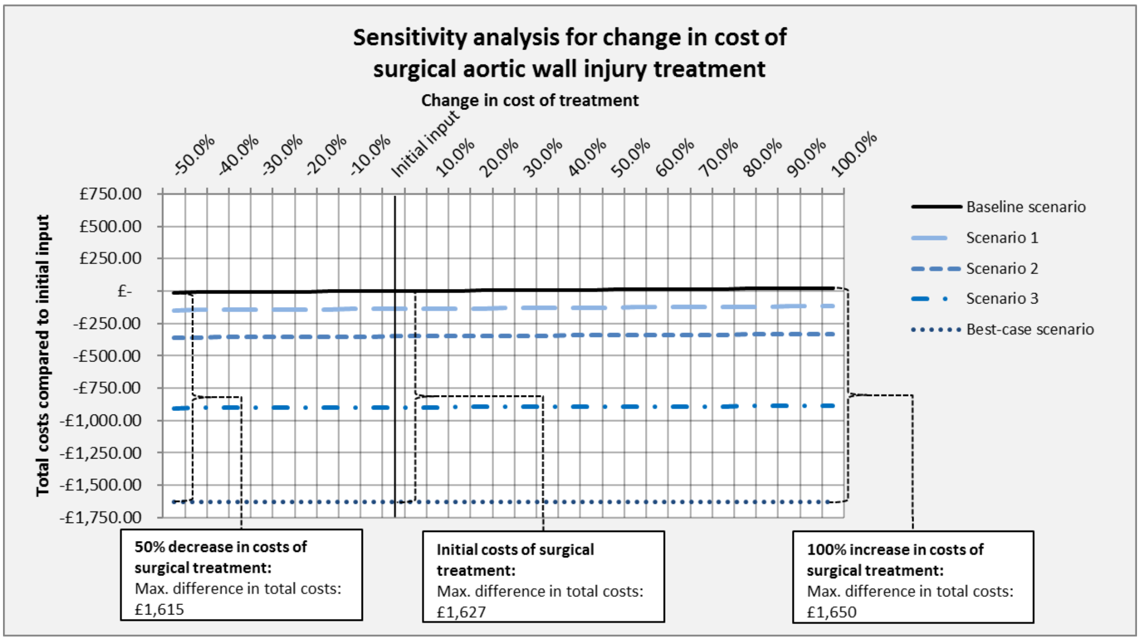

Figure A 5: Sensitivity analysis for cost of surgical CoA repair

The diagram shows the relationship between varying costs for surgical CoA repair (horizontal axis) and expected total costs compared to the initial treatment cost (vertical axis) in each of the four scenarios, as well as the baseline scenario. Varying values of surgical repair cost are displayed relative to the initial input of £7498.

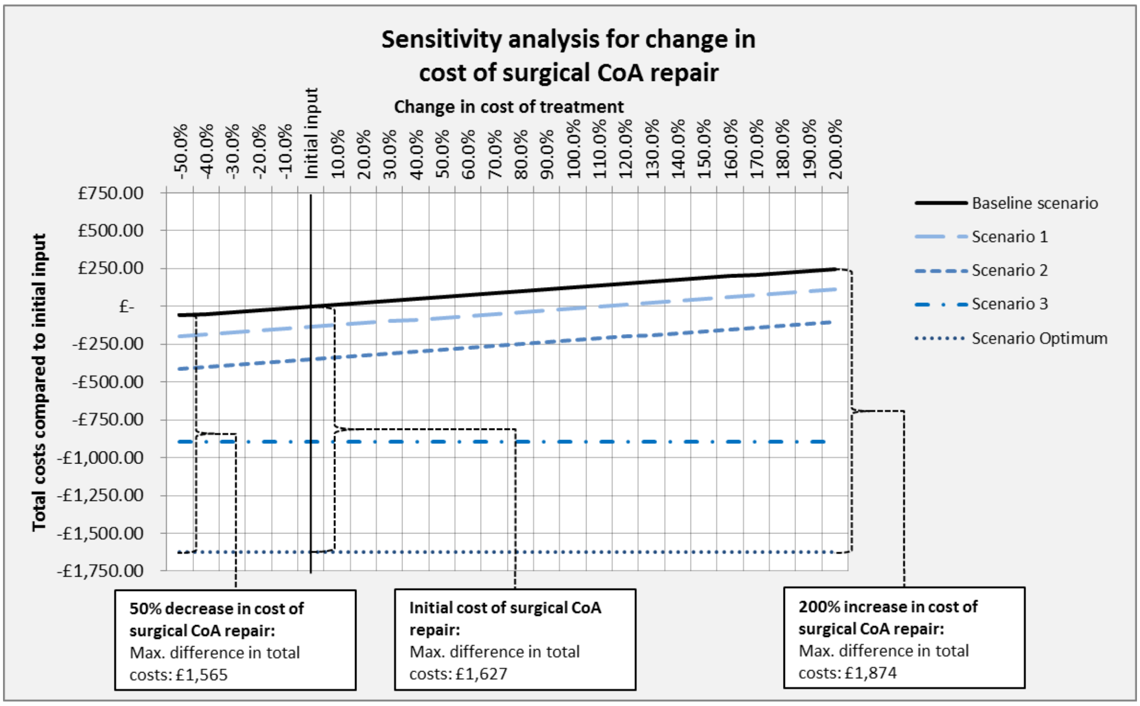

Figure A 6: Sensitivity analysis for cost of imaging

The diagram shows the relationship between varying costs for follow-up imaging (horizontal axis) and expected total costs compared to the initial imaging cost (vertical axis) in each of the four scenarios, as well as the baseline scenario. Varying values of imaging cost are displayed relative to the initial input of £5660.

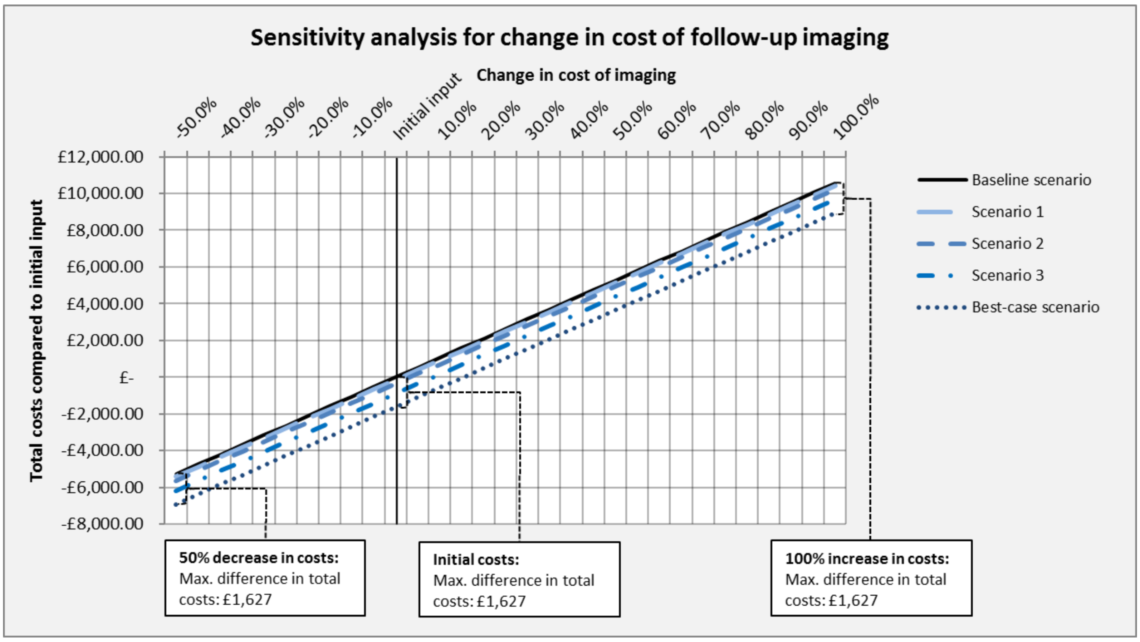

Figure A 7: Sensitivity analysis for cost of hypertension medication

The diagram shows the relationship between varying costs for hypertension medication (horizontal axis) and expected total costs compared to the initial medication cost (vertical axis) in each of the four scenarios, as well as the baseline scenario. Varying values of medication cost are displayed relative to the initial input of £67.5 p.a.

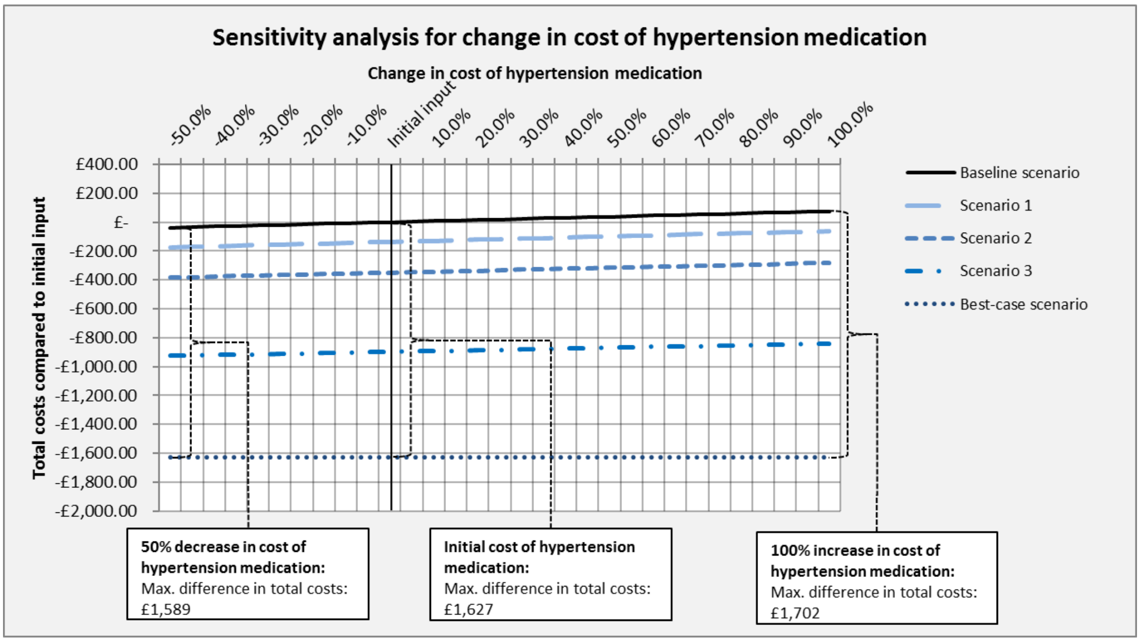

Figure A 8: Sensitivity analysis for stenting success

The diagram shows the relationship between varying probabilities for treatment success (horizontal axis) and expected total costs compared to the initial input value (vertical axis) in Scenarios 1-3, as well as the Baseline scenario. Varying values of treatment success are displayed relative to the initial input (96.7% treatment success).

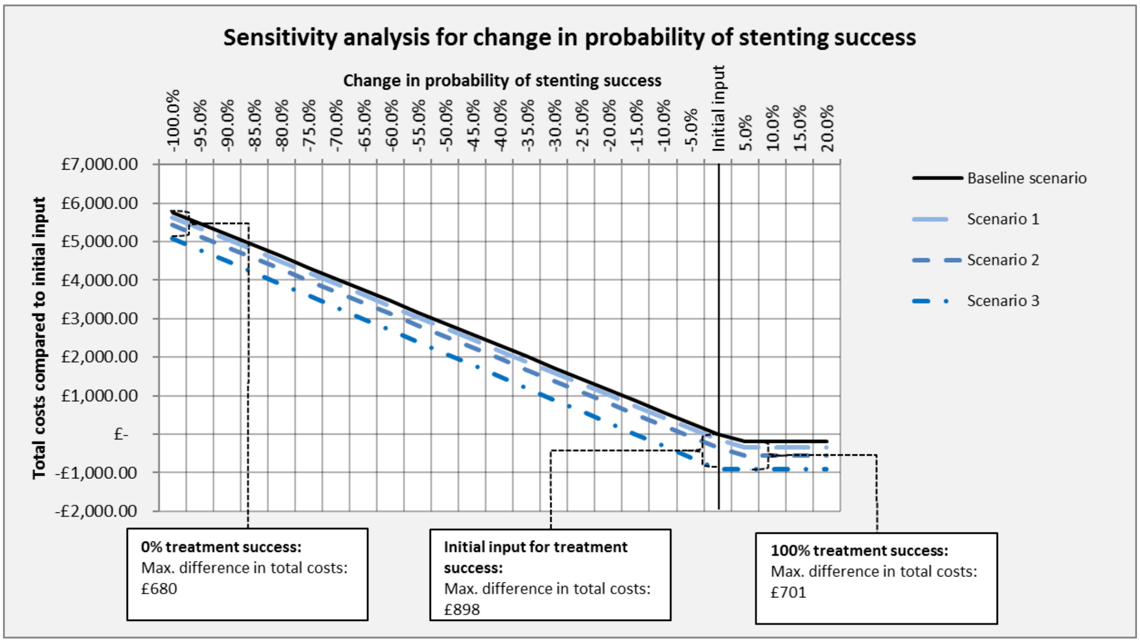

Figure A 9: Sensitivity analysis for aortic wall injury

The diagram shows the relationship between varying probabilities for aortic wall injury after stenting (horizontal axis) and expected total costs compared to the initial input value (vertical axis) in Scenarios 1-3, as well as the Baseline scenario. Varying values of the probability are displayed relative to the initial input (0.8%).

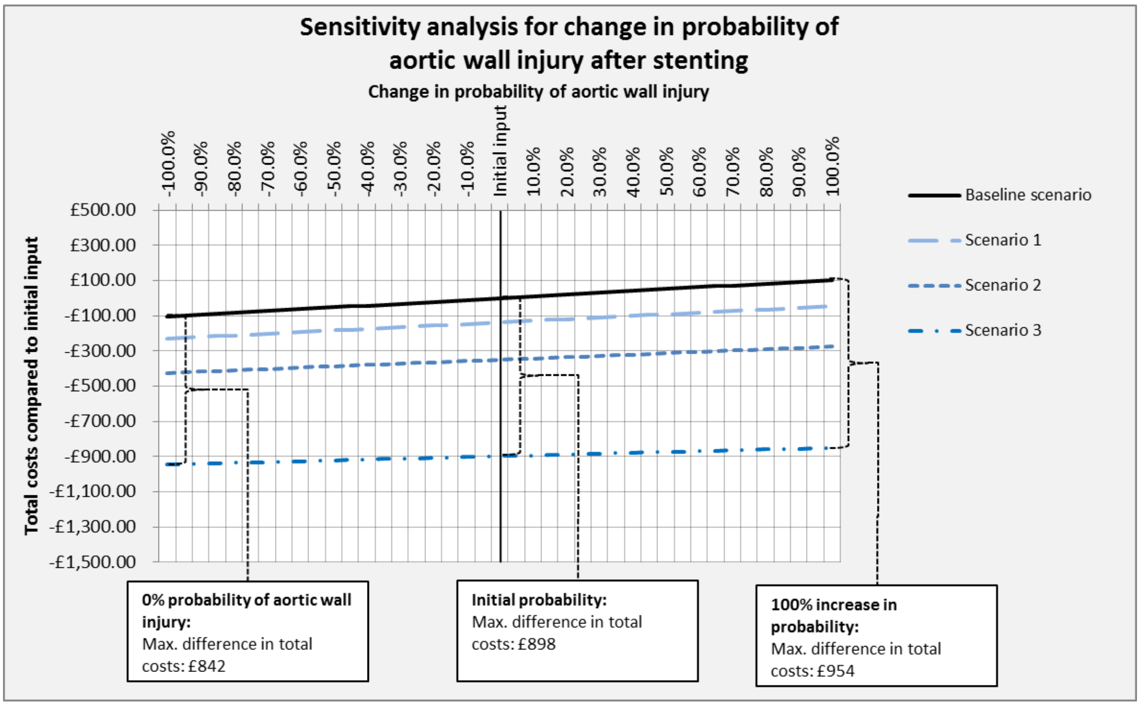

Figure A 10: Sensitivity analysis for follow-up reinterventions

The diagram shows the relationship between varying probabilities for reinterventions at follow-up (horizontal axis) and expected total costs compared to the initial input value (vertical axis) in Scenarios 1-3, as well as the Baseline scenario. Varying values of reintervention rates are displayed relative to the initial input of 9.1% at short-term and 18.5% at mid-term follow-up.

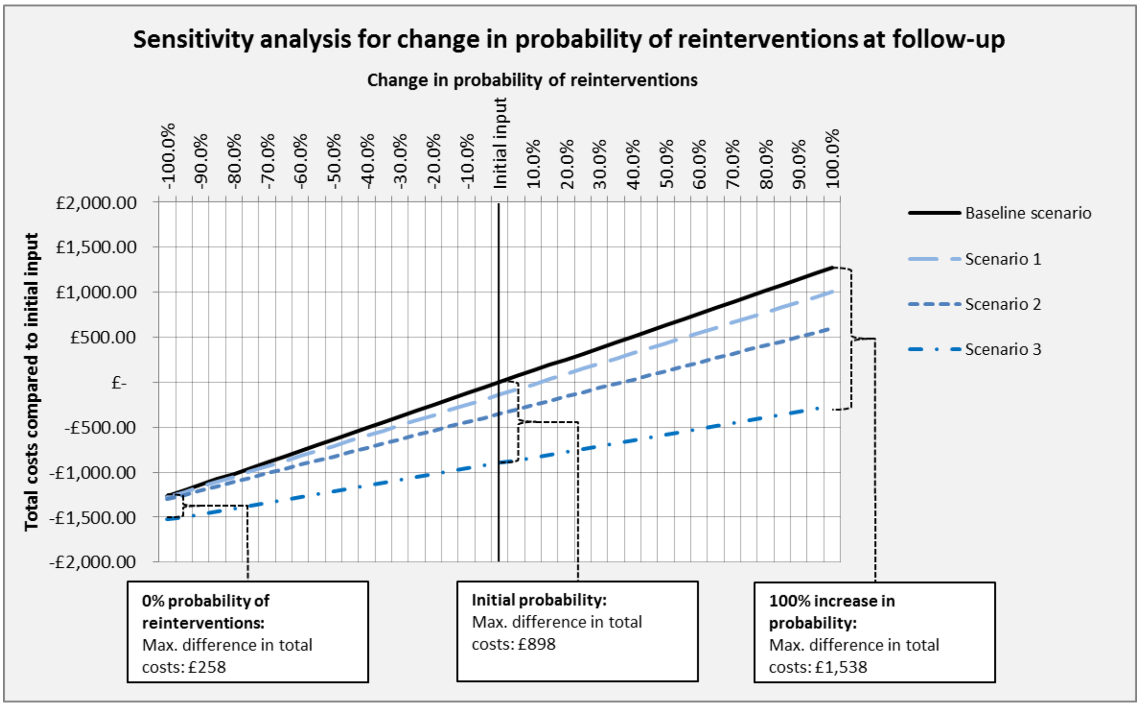

Figure A 11: Sensitivity analysis for hypertension at follow-up

The diagram shows the relationship between varying proportions of patients with hypertension at follow-up (horizontal axis) and expected total costs compared to the initial input value (vertical axis) in Scenarios 1-3, as well as the Baseline scenario. Varying values of proportions are displayed relative to the initial input of 43% at short-term and 39% at mid-term follow-up.

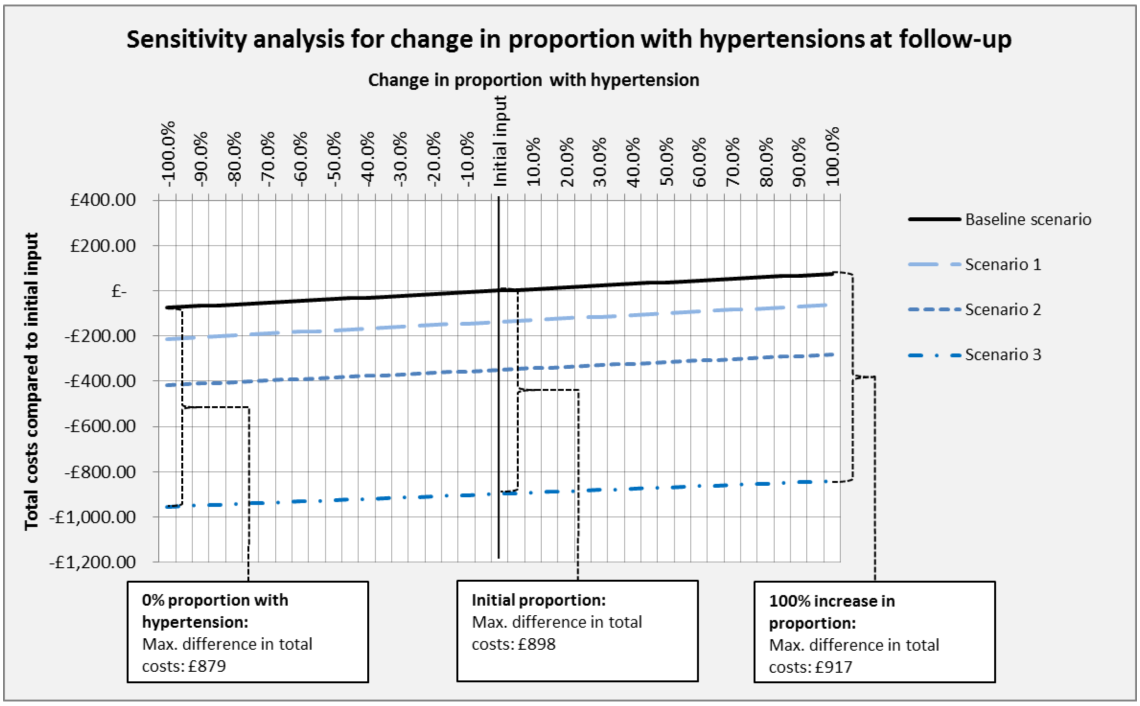

Figure A 12: Sensitivity analysis for interventional aortic wall injury treatment

The diagram shows the relationship between varying probabilities for interventional treatment of aortic wall injury (as opposed to surgery) (horizontal axis) and expected total costs compared to the initial input value (vertical axis) in Scenarios 1-3, as well as the Baseline scenario. Varying values of the probability are displayed relative to the initial input of 90%.

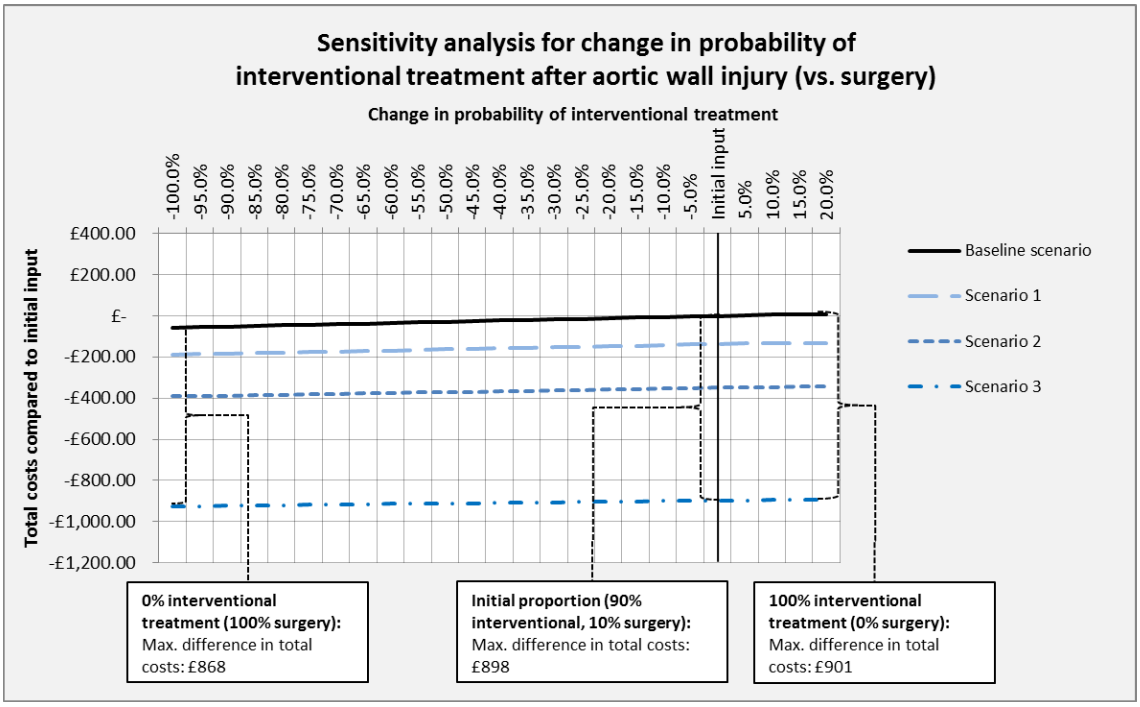

Figure A 13: Sensitivity analysis for repeat stenting

The diagram shows the relationship between varying probabilities for repeat stenting (as opposed to surgery) after unsuccessful initial stenting (horizontal axis) and expected total costs compared to the initial input value (vertical axis) in Scenarios 1-3, as well as the Baseline scenario. Varying values of the probability are displayed relative to the initial input of 50%%.

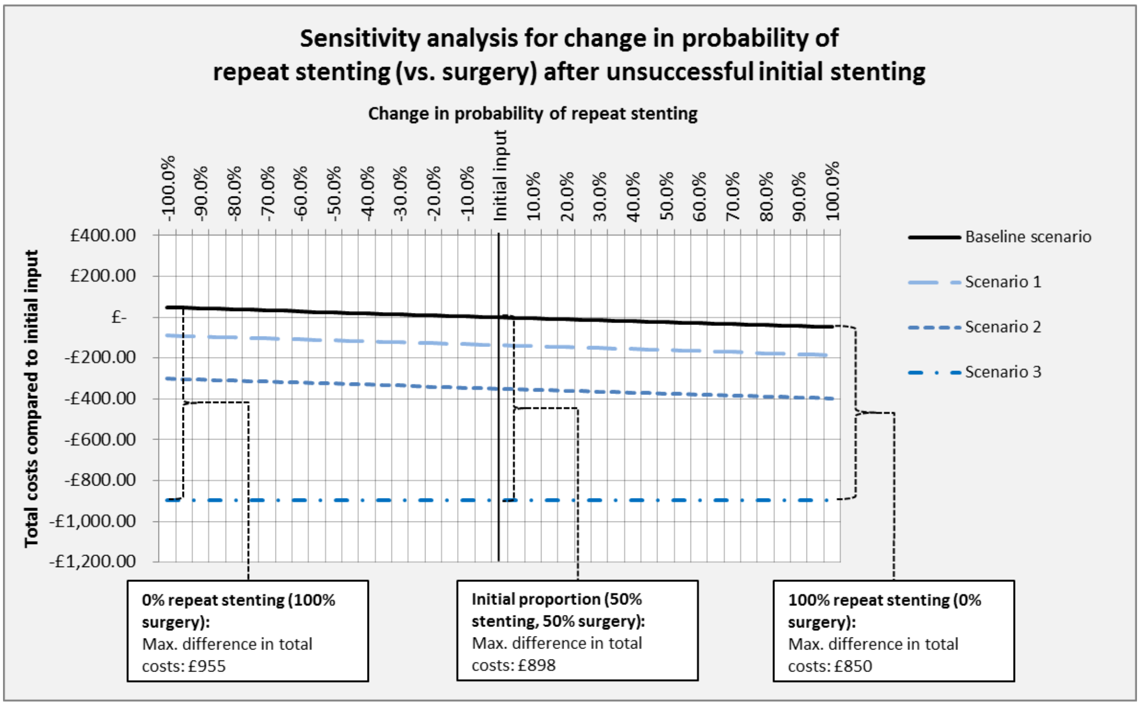

Figure A 14: Sensitivity analysis for aortic wall reinterventions at follow-up

The diagram shows the relationship between varying probabilities for reinterventions at follow-up due to aortic wall injury (as opposed to re-CoA) (horizontal axis) and expected total costs compared to the initial input value (vertical axis) in Scenarios 1-3, as well as the Baseline scenario. Varying values of probabilities are displayed relative to the initial input of 10% at short-term and 5% at mid-term follow-up.

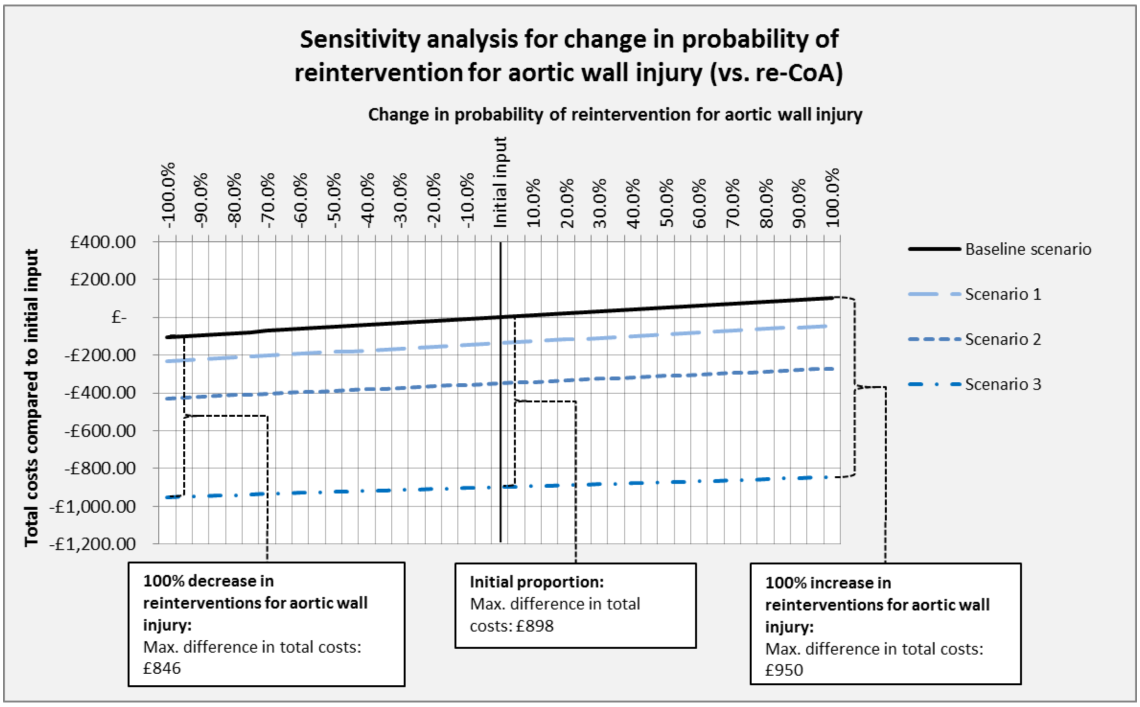

References

1. Dellborg M, Berntsson C, Furenas E, Jivegard L, Liden H, Pettersson J, et al. Surgical or endovascular intervention for aortic coarctation in adult native, residual or re-coarctation? [Internet]. Gothenburg, Sweden: Sahlgrenska Universitetssjukhuset, HTA - centrum; 2014. Report No.: 2014:73. Available from: https://www2.sahlgrenska.se/upload/SU/HTA-centrum/HTA-rapporter/HTA-report%20%20Surgical%20or%20endovascular%20intervention%20for%20aortic%20coarctation%20in%20adult%20native,%20residual%20or%20re-coarctation%202014-08-11.pdf

2. George JC, Shim D, Bucuvalas JC, Immerman E, Manning PB, Pearl JM, et al. Cost-effectiveness of coarctation repair strategies: endovascular stenting versus surgery. Pediatr. Cardiol. 2003;24:544–7.
